# ATP Facilitates Staphylococcal Enterotoxin O Induced Neutrophil IL-1β Secretion *via* NLRP3 Inflammasome Dependent Pathways

**DOI:** 10.3389/fimmu.2021.649235

**Published:** 2021-05-04

**Authors:** Fengqing Hou, Lianci Peng, Jiali Jiang, Tingting Chen, Dongyi Xu, Qingyuan Huang, Chao Ye, Yuanyi Peng, Dong-Liang Hu, Rendong Fang

**Affiliations:** ^1^ Joint International Research Laboratory of Animal Health and Animal Food Safety, College of Veterinary Medicine, Southwest University, Chongqing, China; ^2^ Chongqing Animal Disease Prevention and Control Center, Chongqing, China; ^3^ Department of Zoonoses, School of Veterinary Medicine, Kitasato University, Towada, Japan; ^4^ Immunology Research Center, Medical Research Institute, Southwest University, Chongqing, China

**Keywords:** *Staphylococcus aureus*, staphylococcal enterotoxin O, neutrophils, inflammatory response, NLRP3 inflammasome

## Abstract

*Staphylococcus aureus* (*S. aureus*) is an important zoonotic food-borne pathogen causing severe invasive infections, such as sepsis, pneumonia, food poisoning, toxic shock syndrome and autoimmune diseases. Staphylococcal enterotoxin O (SEO) is a new type of enterotoxins of *S. aureus* with superantigenic and emetic activity. However, it is still unclear about SEO-induced host inflammatory response. Therefore, the mechanism of SEO-induced interleukin-1β (IL-1β) secretion in mouse neutrophils was investigated in this study. Our results showed that recombinant SEO had superantigenic activity with high level of gamma interferon (IFN-γ) production in mouse spleen cells and induced inflammatory cytokines expression including IL-1α, IL-1β, IL-6 and TNF-α in neutrophils under the action of ATP. In addition, SEO-induced IL-1β secretion was dependent on activation of Toll like receptor 4 (TLR4), nuclear factor kappa B (NF-κB) and c-jun N-terminal kinase (JNK) signaling pathways. However, SEO-induced IL-1β secretion was abolished in the neutrophils of NLRP3^-/-^ mice compared with those of wild type mice, indicating that activation of NLRP3 inflammasome mediated IL-1β secretion during neutrophils stimulation with SEO under the action of ATP. Moreover, this process of SEO+ATP-induced IL-1β secretion was dependent on potassium (K^+^) efflux. Taken together, our study suggests that activation of TLR4/JNK/NLRP3 inflammasome signaling pathway mediate maturation and secretion of IL-1β and provides a new insight on *S. aureus* virulence factor-induced host immune response.

## Introduction

Staphylococcal enterotoxins (SEs) are superantigenic exotoxins of *S. aureus*, in which are the leading causes of food poisoning (1). SEs belong to the pyrogenic toxin family, which is classified as classic toxins (SEA to SEE), new types of SEs (SEG to SEI, SEK to SET) and SE-like (SEl) toxin (SElJ and SElU to SElY) (2–4). Staphylococcal enterotoxin O (SEO) is a member of the new types of SEs with superantigenic and emetic activity (5, 6). SEs can stimulate the massive proliferation of T cells by directly binding to major histocompatibility complex class II (MHC II) molecules of antigen presenting cells (APCs) and Vβ regions of T cell receptor (TCR) (7–9), leading to the release of inflammatory cytokines including IL-2, TNF-α and IL-6 (1, 10). It has been shown that SEA, SEB and SEC induced production of IL-1β and TNF-α in human alveolar macrophages and peripheral blood mononuclear cells (11, 12). However, SEO-induced inflammatory response in the host is still unclear.

Innate immunity including specific immune cells plays an important role of host defense against microbial infection. Neutrophils are considered as one of the first responder cells against invading pathogens. They rely on different pattern recognition receptors (PRRs) including Toll-like receptors (TLRs), nucleotide-binding domain leucine-rich repeat containing receptors (NLRs) and C-type lectin receptors (CLRs) to recognize pathogen-associated molecular patterns (PAMPs) (13–15), resulting in production of inflammatory cytokines, such as IL-1β. IL-1β is an important inflammatory cytokine, playing a critical role in the host defense against pathogenic infection and inflammatory disease. The production of IL-1β needs two steps. Firstly, IL-1β is translated in the cytoplasm as an inactive pro-IL-1β, then cleaved to the bioactive mature IL-1β to be secreted (16–18). It has been shown that activation of the NOD-like receptor family, pyrin domain containing 3 (NLRP3) inflammasome causes activation of caspase-1, leading to IL-1β secretion (19). Although the importance of IL-1β in the host defense against *S. aureus*, less is known about the role of toxins of *S. aureus* such as SEO in IL-1β production.

In this study, we investigated the mechanism of SEO-induced IL-1β in primary mouse peritoneal neutrophils. The results demonstrated that activation of NLRP3 inflammasome and caspase-1 mediates maturation and secretion of IL-1β during neutrophils stimulation with SEO under the action of ATP and this process is dependent on K^+^ efflux. Our study provides a new insight on the host pro-inflammatory immune response against staphylococcal enterotoxin.

## Material and Methods

### Mice

The wild-type (WT) C57BL/6 mice were purchased from Chongqing Academy of Chinese Material Medical, China. NLRP3^-/-^, ASC^-/-^, Caspase-1^-/-^, TLR4^-/-^, TLR2^-/-^ mice were kindly provided by Feng Shao from the NIBS (National Institute of Biological Sciences, Beijing, China). All gene knockout mice were on a C57BL/6 background and maintained in SPF (Specific Pathogen Free) conditions for being used at 7-9 weeks old. All of the animal experiments were approved by The Southwest University Ethics Committee and National Institutes of Health care principles of laboratory animal of China.

### Reagents

Anti-mouse IL-1β Ab was purchased from Bioss (Beijing, China); Anti-caspase-1 (p20) mAb was purchased from AdipoGen (San Diego, CA); Anti-GSDMD Ab was purchased from Abcam (Cambridge, UK); Anti-JNK mAb and anti-phospho-JNK mAb were purchased from Cell Signaling Technology (Danvers, USA); Anti-β-actin Ab, HRP-conjugated goat anti-mouse IgG and goat anti-rabbit IgG were purchased from Beyotime Biotech Co. Ltd (Shanghai, China). ELISA kits of mouse IL-1β, IL-6, IL-1α, IFN-γ and TNFα were purchased from Invitrogen (San Diego, CA).

### Expression and Purification of SEO

Recombinant SEO was obtained using the *Escherichia coli* expression systems. Briefly, the *seo* genes from *S. aureus* strains were amplified by PCR. The PCR primers were SEO/GST+(5’-GCCATGGCTGATATCGGATCCATGTTAAATGTAATATTATTAAT) containing a *Bam*HI site and SEO/GST-(5’-GTGGTGGTGGTGGTGCTCGAGTTATGTAAATAAATAAACATC) containing an *Eco*RI site. The predicted size of the PCR product was 765 bp. The pGEX-6P-1 glutathione S-transferase (GST) gene fusion vector was digested with *Bam*HI and *Eco*RI (Takara, Dalian, China). Then, the *seo* gene fragments were cloned into a pGEX-6P-1 glutathione S-transferase (GST) gene fusion vector. After that, the GST-SEO fusion recombinant proteins were expressed and purified, subsequently the fusion proteins were cleaved by Prescission Protease (Beyotime, Beijing, China) and the GST tag was removed by Glutathione Sepharose 4B affinity colum (GE Healthcare, Sweden) from recombinant SEO as described by Ono HK et al. (5). The protein concentration was determined by the Bradford method (Bio-Rad Laboratories, Richmond, CA, USA). The recombinant protein bands were detected by SDS-PAGE. Endotoxin contamination in the purified SEO proteins was removed by ToxinEraser™ Endotoxin Removal Kit (GenScript, Nanjing, China) and the remained endotoxin was detected by Endotoxin Assay Kit (BIOENDO, Xiamen, China). Finally, polymyxin B sulfate (LPS inhibitor, 100 U/mL) was used to remove contaminated LPS in purified SEO prior to stimulation.

### Superantigenic Activity of Purified SEO

To determine superantigenic activity of purified SEO, mouse spleen cells were obtained aseptically and stimulated with different concentrations of SEO and SEA. Then, 100 U/mL of polymyxin B sulfate (lipopolysaccharide inhibitor) (Sigma-Aldrich, St. Louis, M O) was added for 48 h in 24-well plates. The level of IFN-γ in the culture supernatants were analyzed by ELISA, as described previously (4, 20).

### Neutrophils Isolation and Stimulation *In Vitro*

Mice were injected intraperitoneally with 2-3 mL of 4% thioglycolate medium (Eiken, Tokyo, Japan). After 3-4 h, the mice peritoneal exudate cells were obtained by peritoneal lavage and suspended with RPMI 1640 supplemented with 10% FCS (fetal calf serum) or Opti-MEM (Gibco, USA) as reported previously (21). Then, peritoneal exudate cells were incubated at a density of 2×10^5^ cells/well in 48-well plates. The suspended neutrophils were stimulated with different concentrations of SEO (0.01 μg/mL, 0.1 μg/mL, 1 μg/mL) and incubated at 37°C with 5% CO_2_ for 3 h. After stimulation, 2 mM ATP as second signal to produce IL-1β was added to the cells and incubated for an additional 9 h.

### ELISA

Neutrophils were stimulated with different concentrations of SEO (0.01 μg/mL, 0.1 μg/mL, 1 μg/mL) at 37°C with 5% CO_2_ for 3 h. Then, 2 mM ATP was added to the cells and incubated for an additional 9 h. The culture supernatants were collected after 12 h incubation. Levels of cytokines in all the culture supernatants were analyzed by ELISA according to the manufacturer’s protocols (Invitrogen, USA).

### Real-Time Quantitative PCR (RT-qPCR)

The neutrophils were stimulated with SEO (1 μg/mL) for 4 and 8 h. After stimulation, total RNA was extracted by using the RNA Pre-Pure kit (Tiangen, Beijing, China). Complementary DNA was then synthesized by PrimeScript RT reagent Kit (TaKaRa, Dalian, China) according to the manufacturer’s instructions. Quantitative real-time RT-PCR was carried out using SsoFast Eva Green Super-Mix (Bio-Rad, Hercules, CA) and performed on a Bio-Rad CFX 96 instrument. Primers were used as follows: *IL-1β* forward 5’-GAAATGCCACCTTTTGACAGTG and reverse 5’-TGG ATGCTCTCATCAGGACAG, *β-actin* forward 5’-TGGAATCCTGTGGCATCCATGAAAC and reverse 5’-TAAAACGCAGCTCAGTAACAGTCCG, *NLRP3* forward 5’-CTTTCTGGACTCTGACCGGG and reverse 5’-CTCCCATTCTGGCTCTTCCC. The relative expression was analyzed against the expression level of β-actin.

### Western Blot

Neutrophils were cultured with opti-MEM (Gibco, USA) in 12-well plates (5 ×10^5^ cells/well) for 12 h and then were stimulated with 1μg/mL SEO for 3 h. Next, 2 mM ATP (Sigma-Aldrich, St. Louis, MO) was added to the cells and incubated for an additional 9 h. After stimulation, the culture supernatants and cell lysates were collected as previously described (21). Protein concentrations were measured using Bradford protein Assay Kit (Beyotime, Beijing, China). Then proteins were subjected to 12% SDS-PAGE and transferred onto a polyvinylidene difluoride (PVDF) membrane by electroblotting. The membranes were blocked with 5% nonfat dry milk. Then, the membranes were immunoblotted with anti-IL-1β Ab (Bioss, Beijing, China), anti-Caspase1-p20 Ab (AdipoGen, USA), anti-GSDMD Ab (Abcam, Cambridge, UK), anti-JNK mAb (Cell signaling Technology, Danvers, USA), anti-phospho-JNK mAb (Cell Signaling Technology, Danvers, USA), streptavidin-horseradish peroxidase (HRP)-conjugated goat anti-rabbit IgG (H+L) antibody, HRP-conjugated goat anti-mouse IgG (H+L) antibody (Beyotime, Beijing, China). β-actin was employed as a loading control for the cell lysates. Finally, the distinct protein bands were detected by ECL detection reagent (Beyotime, Beijing, China).

### Statistical Analysis

Statistical analysis were performed using GraphPad Prism software v6 (San Diego, CA). For comparisons between two groups, Student’s t-test was used to analyze the significant differences. For multigroup comparisons, ANOVA and the Bonferroni *post hoc* test were used. Statistical significance was determined as *P* value, *P < 0.05, **P < 0.01.

## Results

### Identification of Superantigenic Activity of Recombinant SEO

**Figure 1:**
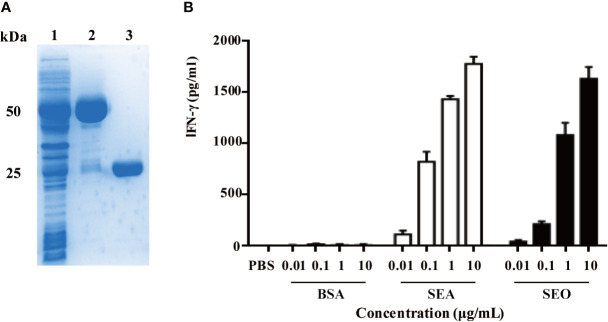
Recombinant expression of SEO and identification of superantigenic activity of SEO. **(A)** SDS-PAGE analysis of purified SEO. Lane 1, supernatants of *Escherichia coli* encoding glutathione S-transferase (GST)-SEO; Lane2, purified GST-SEO fusion protein; Lane3, purified SEO. **(B)** Secretion of IFN-γ in mouse spleen cells in response to stimulation with 0.01 to 10 μg/mL of SEO. Positive control cells were stimulated with SEA. Negative control cells were stimulated with BSA.

Recombinant SEO were obtained using the *Escherichia coli* expression system. The biological and superantigenic activities of purified recombinant SEO protein were identified. SDS-PAGE analysis showed that the band of purified SEO protein located at 25~30 kDa (**Figure 1A**). The endotoxin contamination in the purified SEO proteins was not detected (less than 0.4 ng/mL), indicating the high purity of SEO. To identify the superantigenic activity of SEO, SEO-induced gamma interferon (IFN-γ) in the supernatant of mouse spleen cells was determined by ELISA. Another enterotoxin SEA and BSA were used as positive and negative control, respectively. Both of purified SEO and SEA stimulated spleen cells to produce IFN-γ in a dose dependent manner compared with the BSA (**Figure 1B**). These results indicate that the recombinant SEO remains superantigenic activity.

### SEO Induces Inflammatory Cytokine Expression in Neutrophils Under the Action of ATP

**Figure 2:**
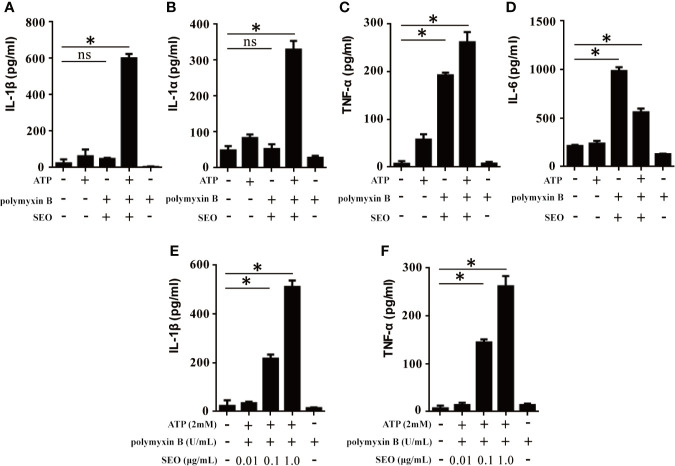
SEO+ATP-induced proinflammatory cytokine secretion in neutrophils. Neutrophils were stimulated with 1 μg/mL of SEO. Then, 2 mM ATP was added to cells and incubated for an additional 9 h. The secretion levels of IL-1β **(A)**, IL-1α **(B)**, TNF-α **(C)** and IL-6 **(D)** in the supernatants were measured by ELISA. Neutrophils from C57BL/6 WT mice were treated with 0.01 μg/mL, 0.1 μg/mL and 1 μg/mL recombinant SEO protein for 3 h. Then, 2 mM ATP was added to cells and incubated for an additional 9 h. IL-1β **(E)** and TNF-α **(F)** secretion in the supernatants were tested by ELISA. All of the experiments were repeated three times. Statistical significance was determined by student’s *t*-test, *p < 0.05; ns, no significance.

To investigate SEO-induced immune response, mice peritoneal neutrophils were stimulated with SEO and the levels of cytokines in the cell culture supernatants were determined by ELISA. The results showed that only SEO did not induce secretion of IL-1β and IL-1α in neutrophils (**Figures 2A, B**), but significantly induced secretion of TNF-α and IL-6 (**Figures 2C, D**), suggesting that secretion of different cytokines involves in different signaling pathway. However, SEO induced secretion of IL-1β and TNF-α in a dose-dependent manner under the action of ATP (**Figures 2E, F**), indicating that SEO-induced IL-1β secretion requires ATP as the second stimulation signal.

It has been reported that LPS+ATP can activate inflammasomes, resulting in IL-1β secretion. To exclude the effect of LPS contamination in SEO+ATP-induced cytokines secretion, we further analyzed the cytokines secretion induced by different concentrations of LPS+ATP. The results showed that the concentration of LPS less than 0.5 ng/mL did not induce the production of IL-1β and TNF-α (**Supplementary Figures 1A, B**). In our study, the purified SEO proteins contained less than 0.4 ng/mL LPS, indicating that the expression of cytokine induced by SEO+ATP is due to the priming of SEO itself, rather than the LPS contamination.

### TLR4 and JNK Signaling Pathways Are Involved in the IL-1β Secretion During Neutrophils Stimulation With SEO+ATP

**Figure 3:**
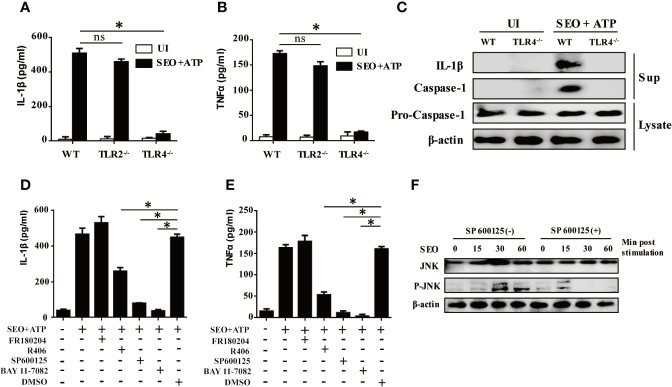
TLR4 and JNK signaling pathways are involved in IL-1β secretion during neutrophils stimulation with SEO+ATP. Neutrophils from C57BL/6 WT, TLR2^-/-^ and TLR4^-/-^ mice were stimulated with 1 μg/mL SEO for 3 h. Then, 2 mM ATP was added to cells, and incubated for an additional 9 h. The supernatants and cell lysates were collected. The secretion levels of IL-1β **(A)** and TNF-α **(B)** were determined by ELISA. Expression of IL-1β (p17), caspase-1 (p20) and pro-caspase-1(p45) in stimulated neutrophils were tested by western blot **(C)**. Neutrophils from C57BL/6 WT were pretreated with the NF-κB inhibitor BAY11-7082 (50μM), JNK inhibitor SP600125 (40μM), ERK inhibitor FR180204 (10μM) and SYK inhibitor R406 (1μM) for 1 h prior to stimulation with SEO. The secretion levels of IL-1β **(D)** and TNFα **(E)** in the culture supernatants were determined by ELISA. Phosphorylated JNK levels were detected by western blot **(F)**. Data are repeated at least three independent experiments. Statistical significance was determined by student’s *t*-test, *p < 0.05; ns, no significance.

It has been reported that release of inflammatory cytokines requires activation of TLRs. To evaluate the role of TLR2, TLR4 in IL-1β and TNF-α secretion upon stimulation with SEO+ATP, we determined expression level of IL-1β and TNF-α in SEO+ATP-stimulated neutrophils of WT C57BL/6, TLR2^-/-^ and TLR4^-/-^ mice by ELISA. The results showed that the ATP-mediated secretion of IL-1β and TNF-α was significantly lower in response to SEO in neutrophils of TLR4^-/-^ mice than those of WT mice (**Figures 3A, B**), but it was not affected in TLR2^-/-^ mice neutrophils (**Figures 3A, B**). Furthermore, western blot analysis showed that the mature form of IL-1β (p17) and caspase-1 (p20) was only detected in cell culture supernatant from SEO+ATP-stimulated neutrophils of WT mice but not in SEO+ATP-stimulated neutrophils of TLR4^-/-^ mice (**Figure 3C**). However, pro-caspase-1 was detected in both cell lysates of SEO+ATP-stimulated neutrophils of WT and TLR4^-/-^ mice (**Figure 3C**). These results indicated that TLR4, but not TLR2, involves in ATP+SEO-induced IL-1β secretion in neutrophils.

Activation of TLRs triggers different intracellular signaling pathways, of which the NF-κB, p38MAPK, JNK and ERK pathways have been reported to play important roles in this process. To investigate the effect of these signaling pathways associated with IL-1β secretion in SEO+ATP-stimulated neutrophils, neutrophils were pre-treated with inhibitors of different signaling pathways (NF-κB inhibitor BAY11-7082, JNK inhibitor SP600125, ERK inhibitor FR180204, SYK inhibitor R406) 1h prior to stimulation with SEO+ATP. After treatment, cells were stimulated with SEO+ATP and the levels of cytokines secretion were determined by ELISA. The results showed that both of NF-κB and JNK inhibitors treatment blocked secretion of IL-1β and TNF-α secretion above baseline levels in SEO+ATP-stimulated neutrophils (**Figures 3D, E**). Treatment with SYK inhibitor reduced secretion of IL-1β and TNF-α in SEO+ATP-stimulated neutrophils (**Figures 3D, E**). ERK inhibitor did not affect the secretion of IL-1β and TNF-α (**Figures 3D, E**). Furthermore, SEO-induced phosphorylation of JNK was observed by western blot after 30 min stimulation but JNK inhibitor abolished SEO-induced phosphorylation of JNK (**Figure 3F**), indicating SEO-induced activation of JNK signaling pathway. Together all, these results demonstrated that NF-κB, JNK and SYK signaling pathways play the key roles in the production of IL-1β in SEO+ATP-stimulated neutrophils.

### NLRP3 Inflammasome Mediates IL-1β Maturation and Secretion During Neutrophils Stimulation With SEO+ATP

**Figure 4:**
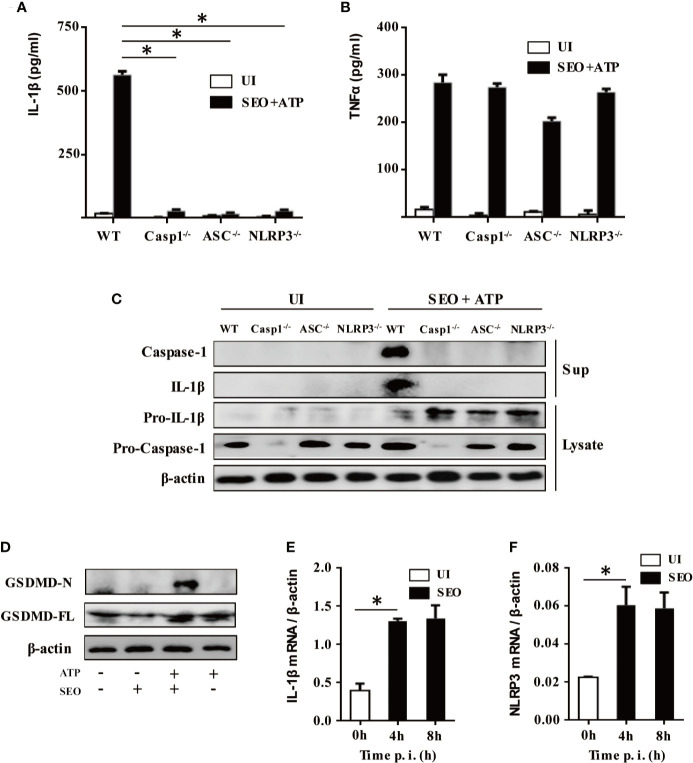
NLRP3 inflammasome mediates IL-1β maturation and secretion during neutrophils stimulation with SEO+ATP. Neutrophils from C57BL/6 WT, NLRP3^-/-^, ASC^-/-^, and Caspase-1^-/-^ mice were stimulated with 1 μg/mL SEO for 3 h. Then, 2 mM ATP was added to cells and incubated for an additional 9 h. The supernatants and cell lysates were collected. The secretion levels of IL-1β **(A)** and TNF-α **(B)** in the supernatants were determined by ELISA. The culture supernatants and cell lysates were analyzed by western blot to detect expression of IL-1β, pro-IL-1β, caspase-1, and pro-caspase-1 **(C)**, GSDMD-N and GSDMD-FL **(D)**. Neutrophils from WT mice were stimulated with 1 μg/mL SEO for 4 h and 8 h. The mRNA expression level of IL-1β **(E)** and NLRP3 **(F)** was analyzed by RT-qPCR. The relative expression was analyzed against the expression level of β-actin. All of the experiments were repeated three times. Statistical significance was determined by student’s *t*-test, *p < 0.05.

To investigate whether NLRP3 inflammasome components were involved in maturation and secretion of IL-1β upon stimulation with SEO+ATP, we tested the secretion level of IL-1β and TNF-α in the cell culture supernatant of SEO+ATP-stimulated neutrophils from C57BL/6 (WT), NLRP3^-/-^, ASC^-/-^ and Caspase-1^-/-^ mice, respectively. The results showed that IL-1β secretion was largely abrogated in SEO+ATP-stimulated neutrophils of NLRP3^-/-^, ASC^-/-^ and Caspase-1^-/-^ mice compared with those of WT mice (**Figure 4A**) while TNF-α secretion was not affected (**Figure 4B**). In addition, western blot analysis also confirmed that mature forms of caspase-1 (p20) and IL-1β (p17) were completely undetectable in SEO+ATP-stimulated neutrophils from NLRP3^-/-^, ASC^-/-^ and Caspase-1^-/-^ mice (**Figure 4C**). Furthermore, Heilig R et al. (22) have been shown that GSDMD is also critical for IL-1β secretion. Therefore, to further assess the activation of GSDMD, neutrophils from WT mice were stimulated with SEO/SEO+ATP. Western blot analysis showed that mature forms of GSDMD-N was detected SEO+ATP-stimulated WT neutrophils while it was not detected in only SEO or ATP-stimulated neutrophils (**Figure 4D**), indicating the priming role of SEO induced immune response.

The activation of NLRP3 pathway needs two signals. The first signal called priming is mainly trigged by bacteria or pathogenic molecules such as lipoprotein and LPS, leading to production of pro-IL-1β, transcriptional and post-translational modification of NLRP3. Therefore, to explore the priming role of SEO, mRNA expression of IL-1β and NLRP3 was determined by RT-qPCR. The results showed that SEO significantly induced the mRNA expression of IL-1β and NLRP3 in neutrophils at 4h and 8h post stimulation (**Figures 4E, F**), indicating that SEO acts as the first signal to activate inflammasomes. Together, these results suggest that IL-1β secretion is mediated by the NLRP3/ASC/Caspase-1 axis during neutrophils stimulation with SEO+ATP. Furthermore, SEO+ATP-induced IL-1β secretion in NLRP3/ASC/Caspase-1-dependent way were also observed in mouse primary macrophages (**Supplementary Figures 2A–D**), which is consistent with the results in neutrophils.

### NLRP3-Mediated IL-1β Secretion During Neutrophils Stimulation With SEO+ATP Is Dependent on K^+^ Efflux

**Figure 5:**
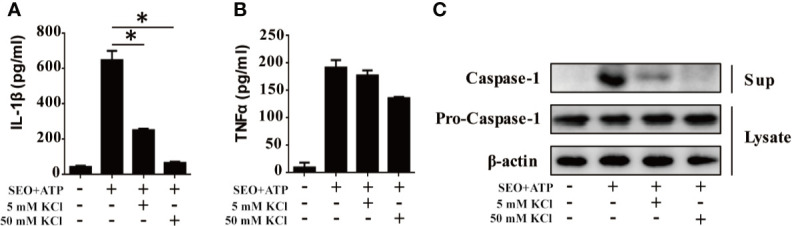
NLRP3-mediated IL-1β secretion during neutrophils stimulation with SEO+ATP is dependent on potassium (K^+^) efflux. Neutrophils from C57BL/6 WT were stimulated with 1 μg/mL SEO under the action of 2 mM ATP for 12 h in 5 mM and 50 mM K^+^ conditions. The secretion levels of IL-1β **(A)** and TNF-α **(B)** were determined by ELISA. Expression of pro-caspase-1 (p45) in cell lysates and mature caspase-1 (p20) in the supernatants were detected by western blot **(C)**. All of the experiments were repeated three times. Statistical significance was determined by student’s *t*-test, *p < 0.05.

K^+^ efflux plays an important role in the assembly and activation of NLRP3 inflammasome (23). Therefore, we investigated the effect of K^+^ efflux on IL-1β secretion during neutrophils stimulation with SEO+ATP. Increased K^+^ concentration in the cell culture medium blocked the secretion of IL-1β in SEO+ATP-stimulated neutrophils (**Figure 5A**), but only slight reduced the secretion of TNF-α (**Figure 5B**). Moreover, we also examined whether the activation of caspase-1 was mediated by K^+^ efflux during neutrophils stimulation with SEO+ATP. Western blot analysis showed that the caspase-1 (p20) mature form was not detected in the high concentration of K^+^ treated group during neutrophils stimulation with SEO+ATP (**Figure 5C**). These results indicated that K^+^ efflux participates in IL-1β secretion during neutrophils stimulation with SEO+ATP.

## Discussion

*S. aureus* causes severe infections in human and animals, such as pneumonia, food poisoning, toxic shock syndrome, and autoimmune diseases (24). During *S. aureus* infection, neutrophils are the first immune cells that are recruited to the site of infection or injury and play a central role in innate immune responses against bacterial infections. The main pathogenesis of *S. aureus* is to produce mass of virulence factors like pore-forming toxins and SEs, which contribute to infection by facilitating tissue attachment, invasion and evading from host immune response (25). It has been reported that some virulence factor of *S. aureus* such as α-Hemolysin (Hlα), toxic shock syndrome toxin-1 (TSST-1) and Panton Valentine leukocidin (PVL) induced innate immune response, but SEO-induced host inflammatory response is still unclear. Therefore, in the present study, we analyzed the biological characteristics of recombinant SEO and investigated the mechanism of IL-1β secretion in SEO-stimulated neutrophils.

Unlike release of many cytokines that requires only a single signal activation such as TLRs, the secretion of IL-1β has been speculated to require at least two specific signals. First, recognition of PAMPs by TLRs induces NF-κB-dependent expression of both pro-IL-1β and NLRP3. Second, danger signals such as ATP and pore-forming toxins etc trigger assembly and activation of NLRP3 inflammasome, subsequently causing cleavage of the pro-IL-1β by the activation of caspase-1 to secret mature IL-1β (26, 27). Our results showed that SEO acted as the first stimulation signal to produce mRNA expression of IL-1β and NLRP3, subsequently ATP as a second signal induced IL-1β secretion. Previous studies have shown that pore-forming toxins such as pneumolysin (28) and suilysin (29) induced IL-1β secretion by activation of TLR4 *via* MyD88-dependent pathway. Recently, we reported that the secretion of IL-1β required the activation of TLR4 in *Pasteurella multocida*-infected macrophages (30). Similarly, in this study, we also found that activation of TLR4 was involved in SEO+ATP-induced IL-1β secretion in neutrophils, indicating that SEO is recognized by TLR4 to induce immune response. However, some research reported that IL-1β secretion was independent of activation of TLR4 in *Streptococcus pneumonia* and *Streptococcus suis*-infected cells *in vitro* (31–33). These results implied that the capacity of pathogen or toxins that induces IL-1β secretion *via* the activation of TLRs is different.

Besides activation of TLRs, activation of some intracellular signaling pathways such as NF-κB and MAPK pathway continues to a signaling cascade, leading to cytokines secretion. Numerous studies have shown that certain kinases such as JNK or Syk can regulate the secretion of IL-1β (34, 35). Recently, we have reported that JNK and Syk signaling pathways is essential for IL-1β secretion in *Streptococcus pneumonia* and *Pasteurella multocida*-infected neutrophils and macrophages (21, 30, 36). Herein, we found that the secretion of IL-1β was dependent on the JNK, NF-κB and Syk pathways during neutrophils stimulation with SEO+ATP, but independent of ERK. In contrast to our study, it was reported that Syk did not affect ATP-induced IL-1β secretion in bone marrow-derived dendritic cells (BMDCs) (37). In addition, it was also reported that *Streptococcus suis*-induced IL-1β secretion in BMDCs was dependent on the NF-κB and ERK signaling pathways, but independent of JNK (33). These results suggest that differential signaling pathways are involved in IL-1β secretion, possibly due to differences in bacteria, toxins and cell types.

It has been reported that IL-1β secretion is dependent on the NLRP3/ASC/Caspase-1 axis in *Salmonella typhimurium* (38), *Staphylococcus aureus* (39), *Listeria monocytogenes* (40) and *Streptococcus pneumonia* (32)-infected macrophages. However, inflammasomes including AIM2, NLRP3 and NLRC4 are also expressed in neutrophils and involved in IL-1β secretion during bacterial infection (41, 42). We previously demonstrated that maturation and secretion of IL-1β require activation of AIM2 and NLRP3 inflammasome in *Streptococcus pneumonia-*infected macrophages and neutrophils (21, 43). Similarly, we found that activation of NLRP3 inflammasome was indispensable for the activation of caspase-1 and secretion of IL-1β in SEO+ATP-stimulated neutrophils and macrophages, indicating that NLRP3 inflammasome plays an important role in controlling IL-1β secretion during neutrophils and macrophages stimulation with SEO+ATP.

NLRP3 inflammasome activation is considered to contain multiple upstream signals such as efflux of potassium ions (K^+^) (44) or chloride ions (Cl^-^) (45), flux of calcium ions (Ca^2+^) (46), lysosomal disruption and mitochondrial dysfunction (47). It has been reported that extracellular ATP triggers activation of the P2X7 receptor, promoting IL-1β maturation *via* K^+^ efflux (26, 48). Similarly, our study showed that NLRP3-mediated IL-1β secretion was dependent on K^+^ efflux in SEO+ATP-stimulated neutrophils. However, upstream signals of NLRP3 inflammasome are complex, most of which are not mutually exclusive. Therefore, more upstream signals of NLRP3 need to be explored in the future study.

Unlike other virulent factors of *S. aureus* such as Hlα and PVL contributing to pathogenesis *in vivo*, SEO as superantigenic toxin did not induce pathogenic diseases. It exerts as the first signaling stimuli to induced activation of NLRP3 *in vitro*, which is similar with another superantigenic toxin TSST-1 of *S. aureus* (49). Hu et al. (50) showed that vaccination with TSST-1 improved protection against *S. aureus* in mice and it did not damage the host. Considering the similar property of superantigenic toxin of *S. aureus*, it is speculated that SEO challenge might play the similar role *in vivo*. However, SEO *and S. aureus* knockout SEO *in vivo* challenge need to be further studied. Our study on SEO-induced immune response *in vitro* provides important information for the development of vaccines against *S. aureus.*

In conclusion, SEO can induce inflammatory cytokines secretion in neutrophils under the action of ATP. SEO+ATP-induced IL-1β secretion in neutrophils is involved in activation of TLR4, NF-κB and JNK. Furthermore, NLRP3 mediates IL-1β secretion *via* K^+^ efflux during neutrophils stimulation with SEO+ATP. Our study provides better understanding on the host pro-inflammatory immune response against superantigenic exotoxin.

## Data Availability Statement

The original contributions presented in the study are included in the article/**Supplementary Material**. Further inquiries can be directed to the corresponding authors.

## Ethics Statement

The animal study was reviewed and approved by Institutional Animal Care and Use Committee of Southwest University (IACUC-2017-0927-06).

## Author Contributions 

FH and LP performed the experiments and drafted the manuscript. JJ, TC, DX, and QH assisted with the experiments, CY and YP helped in analyzing the data. D-LH and RF contributed to the supervision and wrote/corrected the manuscript. All authors contributed to the article and approved the submitted version.

## Funding

This study was supported by the National Key Research and Development Program of China (2018YFD0500500), the National Natural Science Foundation of China (31902256), the Foundation for Innovation Research Group in Chongqing Universities (CXQT20004), and the earmarked fund for China Agriculture Research System (CARS37).

## Conflict of Interest

The authors declare that the research was conducted in the absence of any commercial or financial relationships that could be construed as a potential conflict of interest.

## Supplementary Material

Supplementary Figure 1 LPS+ATP-induced IL-1β and TNFα expression in neutrophils. Neutrophils from C57BL/6 WT mice were stimulated with 0.05 ng/mL, 0.5 ng/mL, 5 ng/mL and 50 ng/mL LPS for 3 h. Then, 2 mM ATP was added to cells and incubated for an additional 9 h. After stimulation, cell supernatants were collected and cytokines were determined by ELISA. The expression of IL-1β **(A)** and TNFα **(B)** in different concentrations of LPS-stimulated neutrophils. Statistical significance was determined by student’s *t*-test, **p* < 0.05; ***p* < 0.01; ns, no significance.

Supplementary Figure 2 SEO+ATP-induced IL-1β secretion in macrophages is dependent on the activation of NLRP3 inflammasome. Macrophages from C57BL/6 WT, NLRP3^-/-^, ASC^-/-^, and Caspase-1^-/-^ mice were stimulated with 1 μg/mL SEO for 3 h. Then, 2 mM ATP was added to cells and incubated for an additional 9 h. After stimulation, cell supernatants were collected and cytokines were determined by ELISA. The secretion levels of IL-1β **(A, C)** and TNF-α **(B, D)** in the supernatants were determined by ELISA. Statistical significance was determined by student’s *t*-test, **p* < 0.05; ns, no significance.
