# Supplementary figures and images for: ATP Facilitates Staphylococcal Enterotoxin O Induced Neutrophil IL-1β Secretion via NLRP3 Inflammasome Dependent Pathways
Source: Front Immunol. 2021 May 4;12:649235. doi: 10.3389/fimmu.2021.649235 (PMC8129502; doi:10.3389/fimmu.2021.649235)

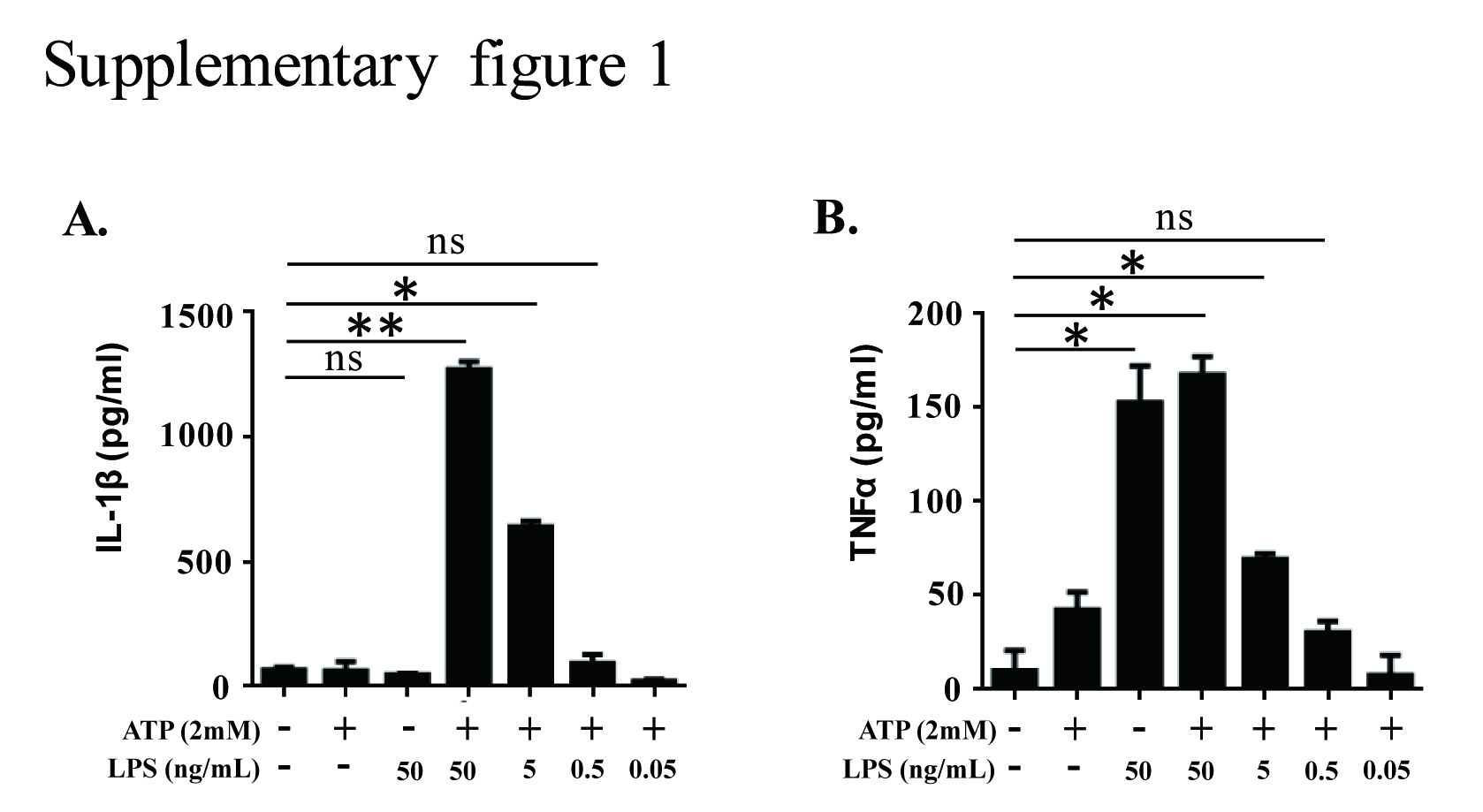

Supplement: Supplementary Figure 1 — LPS+ATP-induced IL-1β and TNFα expression in neutrophils. Neutrophils from C57BL/6 WT mice were stimulated with 0.05 ng/mL, 0.5 ng/mL, 5 ng/mL and 50 ng/mL LPS for 3 h. Then, 2 mM ATP was added to cells and incubated for an additional 9 h. After stimulation, cell supernatants were collected and cytokines were determined by ELISA. The expression of IL-1β (A) and TNFα (B) in different concentrations of LPS-stimulated neutrophils. Statistical significance was determined by student’s t-test, *p < 0.05; **p < 0.01; ns, no significance. [file Image_1.tif]

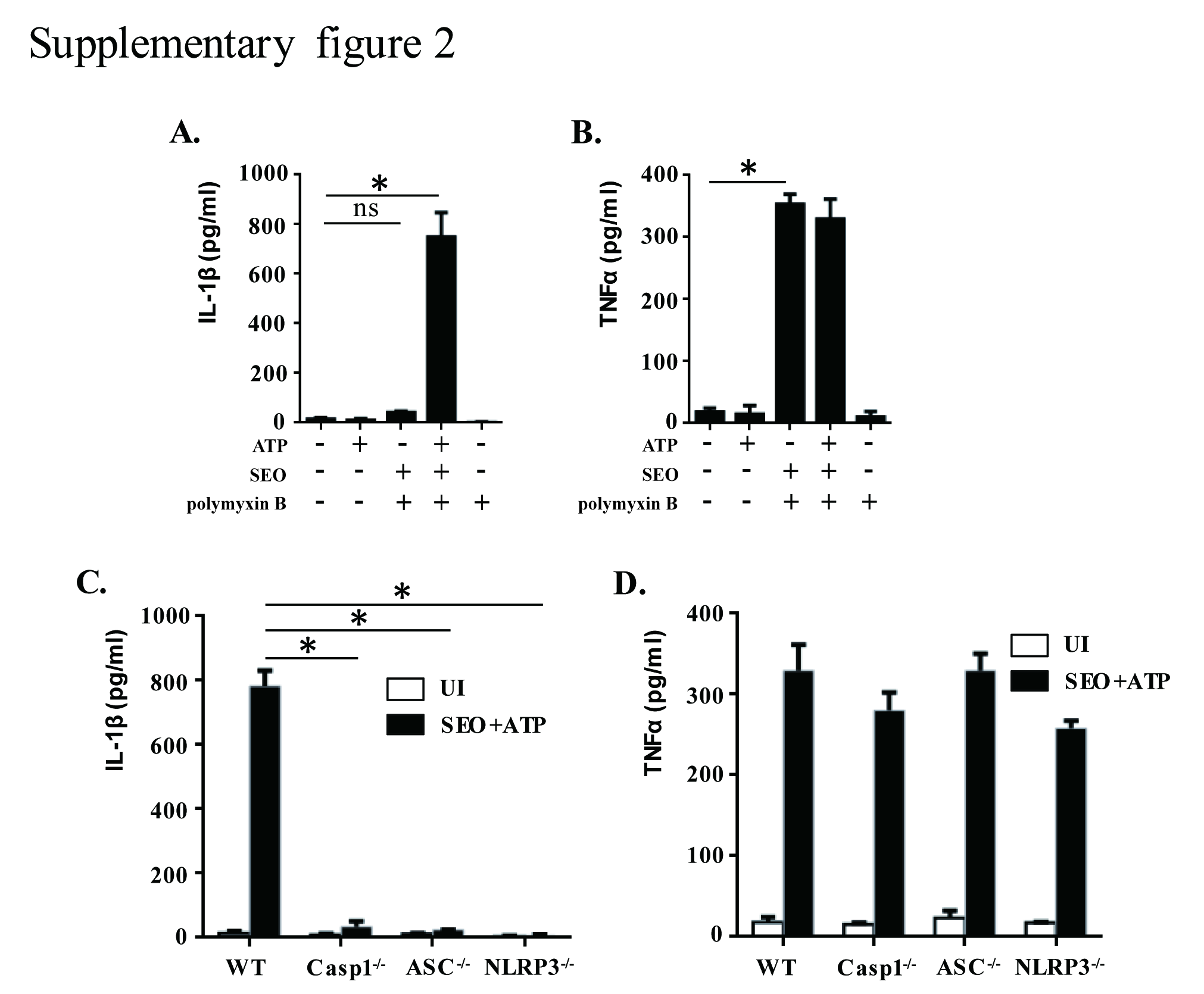

Supplement: Supplementary Figure 2 — SEO+ATP-induced IL-1β secretion in macrophages is dependent on the activation of NLRP3 inflammasome. Macrophages from C57BL/6 WT, NLRP3-/-, ASC-/-, and Caspase-1-/- mice were stimulated with 1 μg/mL SEO for 3 h. Then, 2 mM ATP was added to cells and incubated for an additional 9 h. After stimulation, cell supernatants were collected and cytokines were determined by ELISA. The secretion levels of IL-1β (A, C) and TNF-α (B, D) in the supernatants were determined by ELISA. Statistical significance was determined by student’s t-test, *p < 0.05; ns, no significance. [file Image_2.tif]
