# Supplementary material for: ATP Facilitates Staphylococcal Enterotoxin O Induced Neutrophil IL-1β Secretion via NLRP3 Inflammasome Dependent Pathways
Source: Front Immunol. 2021 May 4;12:649235. doi: 10.3389/fimmu.2021.649235 (PMC8129502; doi:10.3389/fimmu.2021.649235)

Figure 3C

1). IL-1β (p17)

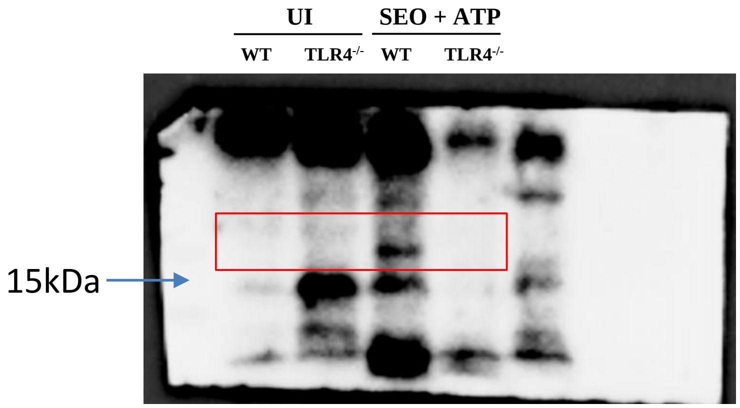

2). Caspase-1 (p20)

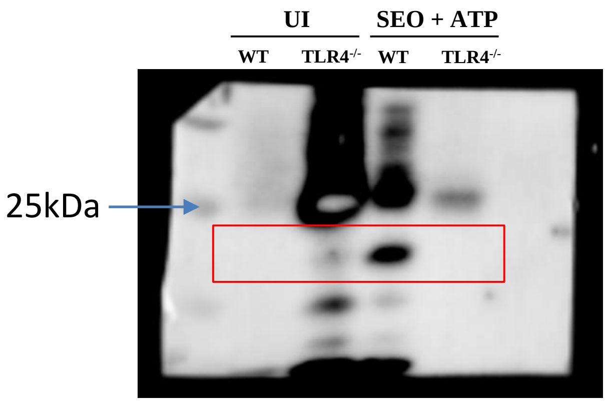

3). Pro-caspase-1 (p45)

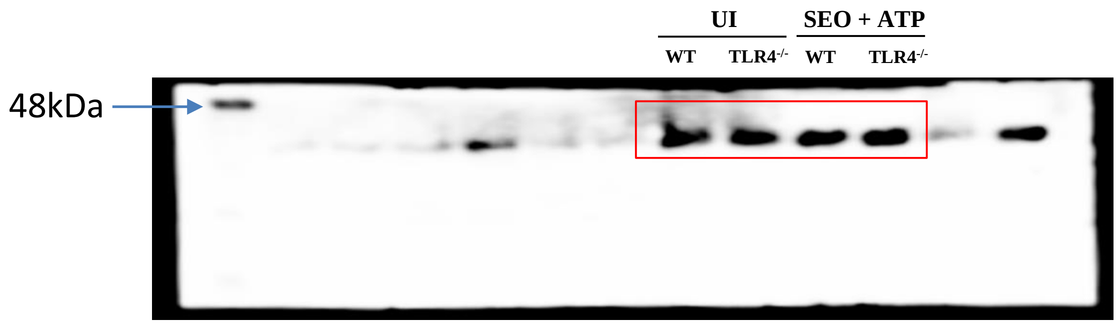

4). β-actin (p42)

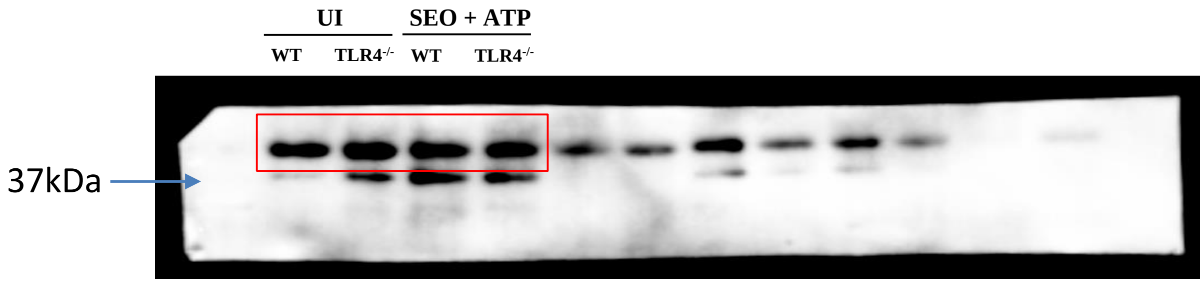

Figure 3F

1).

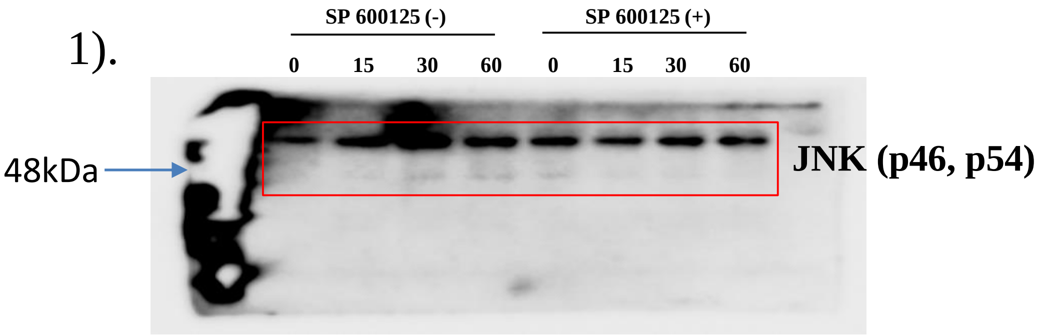

2).

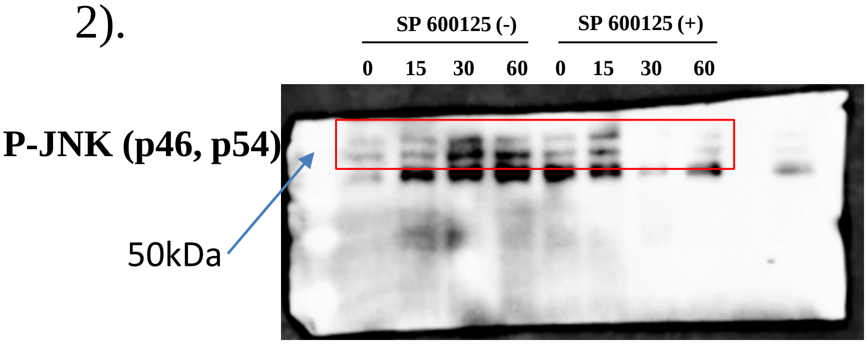

3). β-actin (p42)

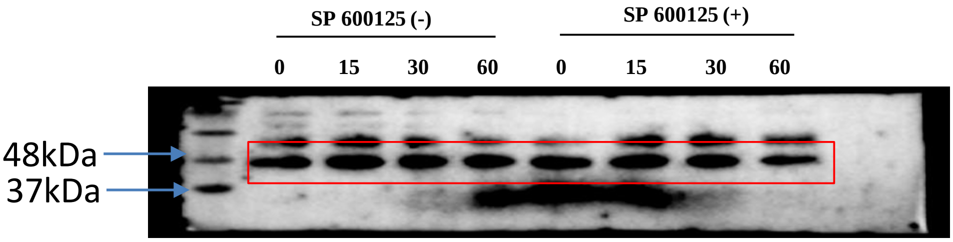

Figure 4C

1). Caspase-1 (p20)

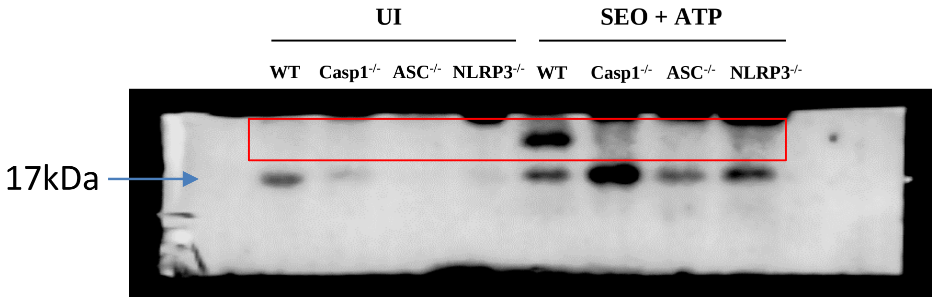

2). IL-1β (p17)

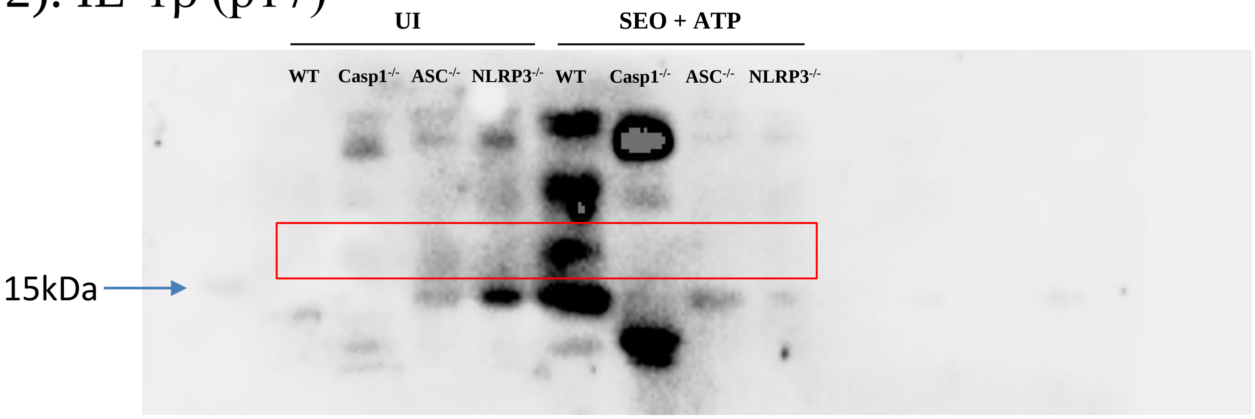

3). Pro-IL-1β (p31)

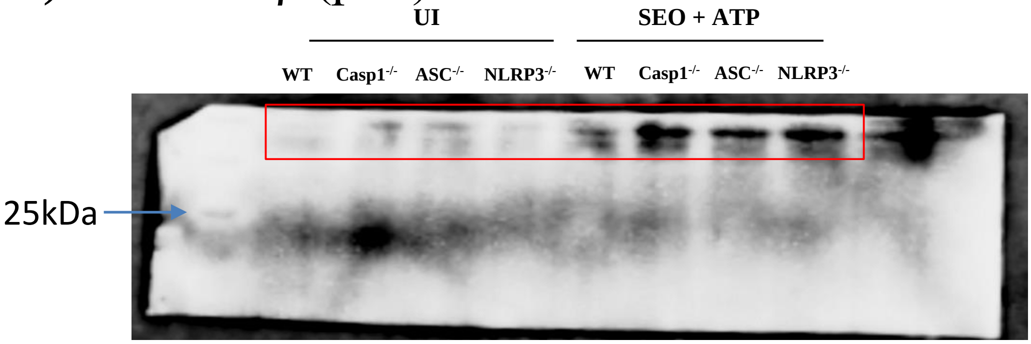

4). Pro-caspase-1 (p45)

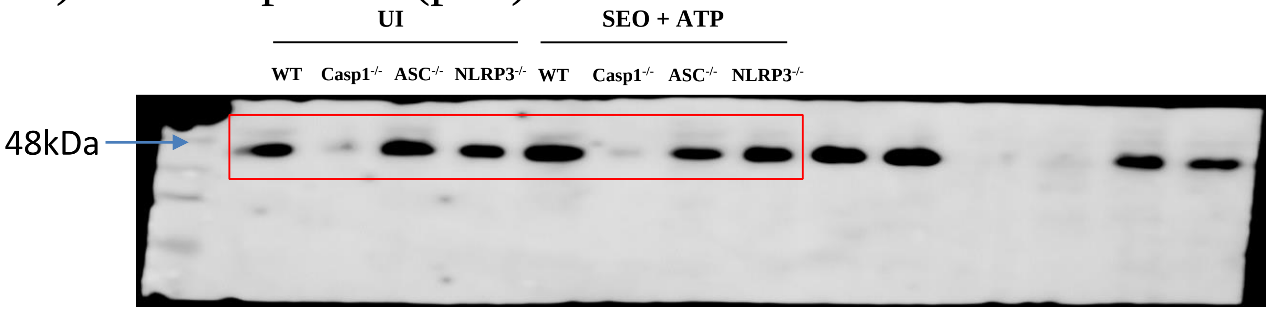

5). β-actin (p42)

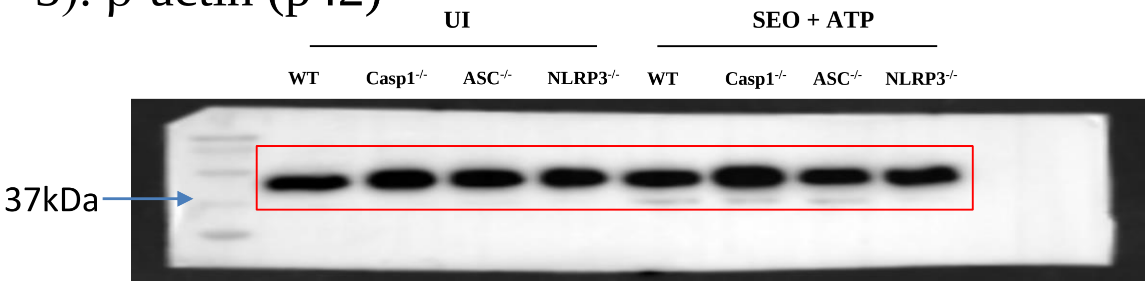

Figure 4D

1). GSDMD-N (p32) GSDMD-FL (p53)

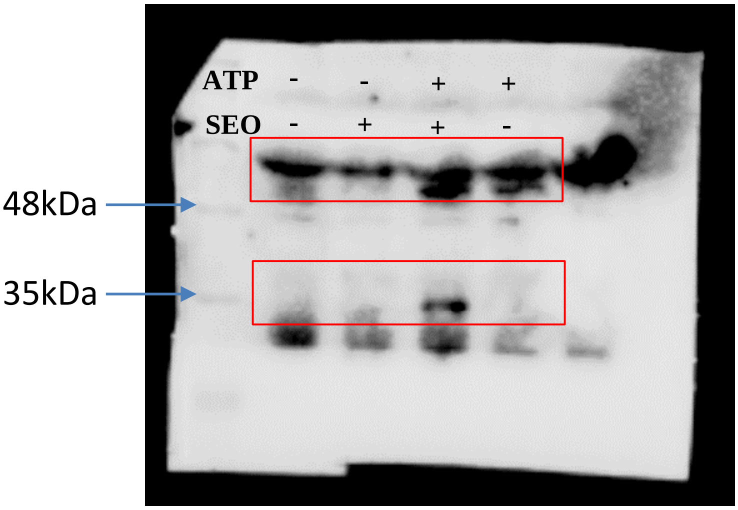

2). β-actin (p42)

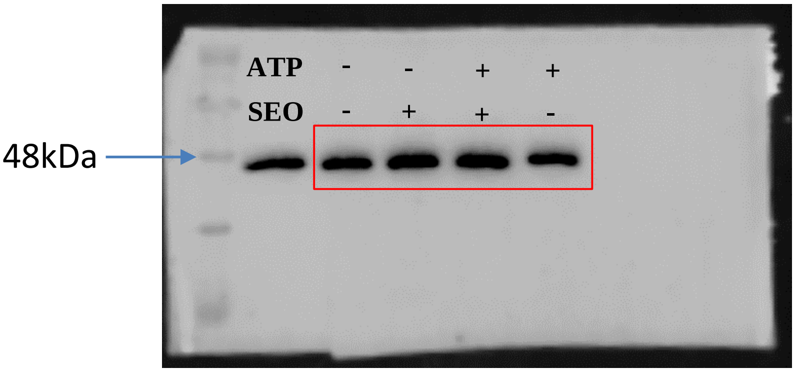

Figure 5C

1). Caspase-1 (p20)

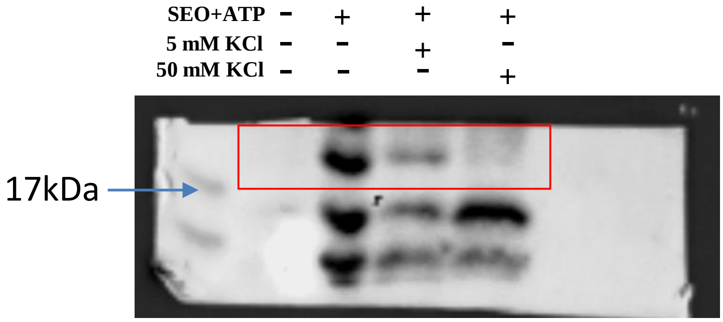

2). Pro-caspase-1 (p45)

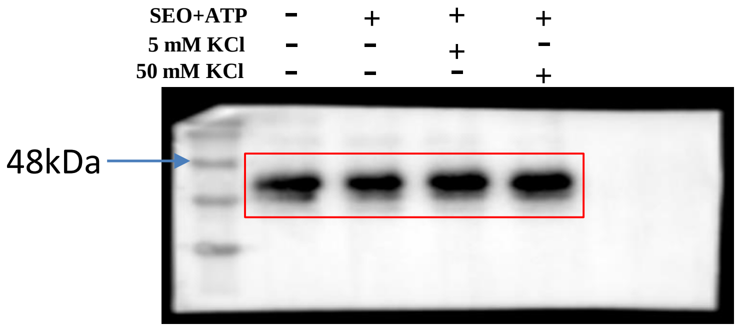

3). β-actin (p42)

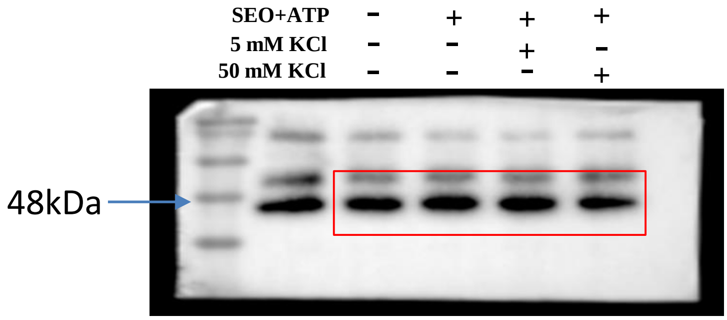

Supplement: Supplementary file 3 [file DataSheet_1.pdf]
